# Freeze–Thaw-Induced Hybrid Porous PVA/PEG Hydrogels with Dynamic Load-Dissipation Capability for Cartilage Substitutes

**DOI:** 10.3390/gels12060494

**Published:** 2026-06-02

**Authors:** Luon Tan Nguyen, Patrick Kai Xuan Lim, Wenjuan Jin, Yanli Zheng, Quang M. N. Phan, Meng Wang, Duc Anh Tran, Y. B. Guo, V. P. W. Shim, Huy-Du Do, Thanh-Tan Nguyen, Hieu Tran-Van, Nga H. N. Do, Hai M. Duong

**Affiliations:** 1 Department of Mechanical Engineering, National University of Singapore, Singapore 117575, Singapore; ntluon.spe@gmail.com (L.T.N.); kaixuanrz@hotmail.com (P.K.X.L.); e1582994@u.nus.edu (W.J.); yanlizheng@u.nus.edu (Y.Z.); quang.phanngominh@u.nus.edu (Q.M.N.P.); e1719839@u.nus.edu (M.W.); tducanh03@u.nus.edu (D.A.T.); mpegy@nus.edu.sg (Y.B.G.); vshim.me@nus.edu.sg (V.P.W.S.); 2 Key Laboratory of Impact and Safety Engineering, Ministry of Education, Ningbo University, Ningbo 315211, China; 3 Laboratory of Biosensors, Faculty of Biology and Biotechnology, University of Science, Ho Chi Minh City 72711, Vietnam; 24c66019@student.hcmus.edu.vn (H.-D.D.); nttan@hcmus.edu.vn (T.-T.N.); tvhieu@hcmus.edu.vn (H.T.-V.); 4 Department of Molecular and Environmental Biotechnology, Faculty of Biology and Biotechnology, University of Science, Ho Chi Minh City 72711, Vietnam; 5 Laboratory of Molecular Biotechnology, University of Science, Ho Chi Minh City 72711, Vietnam; 6 Vietnam National University, Ho Chi Minh City 71308, Vietnam; 7 Institute for Tropical Technology (VITTEP), 57A Truong Quoc Dung Street, Phu Nhuan Ward, Ho Chi Minh City 70073, Vietnam; ngado.spe@gmail.com; 8 Mekong University (MKU), Vinh Long 85216, Vietnam

**Keywords:** hydrogel, polyvinyl alcohol, polyethylene glycol, artificial cartilage, freeze–thaw, dynamic compression

## Abstract

Osteoarthritis is the most prevalent age-related joint disease, yet the limited regenerative capacity of articular cartilage severely constrains spontaneous repair. Here, we present a freeze–thaw polyvinyl alcohol (PVA)/polyethylene glycol (PEG) hydrogel platform featuring a hybrid open–closed macroporous architecture that enables cartilage-mimetic load dissipation for artificial cartilage applications. The hybrid porous structure provides synergistic advantages, where closed pores enhance load-bearing stiffness while open pores facilitate energy dissipation. By systematically tuning polymer composition and processing conditions, clear structure–property relationships among porosity, water content, and mechanical performance are established. An optimized formulation (18 wt.% PVA, 85–124 kDa; 18 wt.% PEG; three freeze–thaw cycles) yields hydrogels with high water content (39.1 ± 7.8 wt.%), high compressive Young’s modulus (3.60 ± 0.67 MPa), and excellent resilience under cyclic loading. Notably, under dynamic compression (2 m/s), a frequently overlooked yet physiologically relevant mechanical property of hydrogels, the materials exhibit nearly twofold enhancement in compressive modulus compared to static conditions, demonstrating pronounced strain-rate-dependent stiffening. Finite element analysis reveals efficient load redistribution across the interconnected porous network, providing mechanistic insight into the observed mechanical robustness. Compared with native cartilage and recently reported hydrogel systems, the developed hydrogels exhibit superior stiffness while maintaining mechanical and structural resilience. In vitro cytotoxicity and direct-contact assays confirm excellent cytocompatibility. These results establish a scalable and cost-effective design strategy for engineering mechanically robust, rate-adaptive hydrogels, advancing the development of next-generation artificial cartilage substitutes.

## 1. Introduction

Articular cartilage is a specialized connective tissue that covers the ends of long bones in diarthrodial joints and exhibits a hierarchical, gradient structure, ranging from a smooth, dense superficial zone to a porous, calcified deep zone [1]. Native cartilage is primarily composed of water, collagen, and glycosaminoglycans, which function synergistically with synovial fluid to provide both high compressive strength (~10 MPa) and an exceptionally low friction coefficient at the bone–cartilage interface (~0.11) [1,2,3,4]. Osteoarthritis is the most prevalent age-related joint disease, characterized by progressive cartilage degradation that leads to pain, stiffness, and reduced mobility [2,5]. Globally, osteoarthritis affects more than 300 million individuals, with approximately 37% of adults over the age of 60 in the United States suffering from knee osteoarthritis [1,5]. Owing to its avascular nature and limited cellularity, articular cartilage possesses minimal intrinsic regenerative capacity, making spontaneous repair of cartilage injuries highly challenging [1]. Consequently, in severe cases, partial or total replacement of the damaged cartilage is often required [5].

Compared with commercial joint replacements (e.g., metal alloys, ceramics, and ultra-high molecular weight polyethylene), hydrogels are widely used in cartilage tissue engineering due to their close resemblance in hydration, microstructure, and mechanical behavior to native biological tissues, as well as their tunable mechanical properties and capacity for drug loading [3,6,7,8]. Among these, polyvinyl alcohol (PVA) hydrogels have been extensively studied due to their excellent biocompatibility, low frictional characteristics, and abundant hydroxyl groups, which impart hydrophilicity and enable a degree of self-healing behavior through reversible hydrogen bonding [1,2,5,9]. A simple and scalable cyclic freeze–thaw (FT) process is commonly employed to fabricate highly porous, spongy, and elastomeric PVA hydrogels, during which polymer chain reticulation is enhanced through liquid–liquid phase separation, hydrogen bonding, and crystallite formation [2,5,10]. The crystalline domains formed within the PVA network contribute significantly to the mechanical strength, elasticity, fatigue resistance, and structural stability of the hydrogel [4,10].

However, pristine PVA hydrogels typically exhibit insufficient mechanical strength for load-bearing cartilage applications [1,2,4]. Based on our preliminary results, PVA hydrogels fabricated via the FT method with PVA concentrations ranging from 16 to 20 wt.% show relatively low compressive Young’s modulus (0.08–0.11 MPa), despite their high water contents (73.2–81.6 wt.%). The mechanical performance of PVA hydrogels can be enhanced through the incorporation of additional crosslinkers, which introduces intermolecular interactions and increases network density [4,11]. In general, two categories of crosslinking strategies are employed in PVA hydrogel fabrication: chemical and physical crosslinking [12,13]. While chemical crosslinkers can significantly improve mechanical properties, they may leave residual reagents that compromise material biocompatibility [2,5]. Consequently, physical crosslinking approaches are considered more attractive due to their high purity, reduced cytotoxic risk, and simplicity of gelation under mild processing conditions [12].

Several physical crosslinkers have been employed to enhance the mechanical performance of PVA hydrogels, including polyethylene glycol (PEG) [4,14], polyacrylic acid [1,5], chitosan [3,6], etc. Among these, PEG is a widely used hydrophilic and biocompatible polymer that has been approved by the U.S. Food and Drug Administration for biomedical applications [15,16]. PEG can interact with PVA chains through hydrogen bonding, promoting the formation of an interconnected polymer network with improved energy dissipation capability and viscoelastic behavior [14]. Furthermore, the hydrophilic ether groups of PEG facilitate water retention and mobility within the hydrogel, thereby improving hydration and reducing frictional resistance [17,18]. Owing to their low cost, excellent biocompatibility, biodegradability in the human body, and immuno-inertness, the combination of PVA and PEG provides a promising material platform for the development of artificial cartilage systems [19,20,21,22].

Liu et al. reported a bioinspired PVA/PEG hydrogel featuring a concrete-like structure composed of PVA particles embedded within a network of PVA/PEG fibers fabricated via the FT method [14]. This hydrogel exhibited outstanding mechanical and tribological properties, including an impressive compressive strength of 29.5 MPa at 86% strain, an excellent tensile strength of 10.5 MPa at 265% strain, and a low friction coefficient of below 0.15 across a range of frequencies and aqueous lubrication conditions. Hao et al. [4] developed a FT-induced PVA hydrogel reinforced with PEG, hyaluronic acid, and collagen. In addition to excellent biocompatibility, the resulting hydrogel demonstrated a high compressive strength (31 MPa), high toughness (1.17 MJ/m^3^), and an exceptionally low friction coefficient (0.01).

In addition to crosslinking chemistry, the morphological architecture of PVA/PEG hydrogels plays a critical role in governing their mechanical performance. For instance, Zhao et al. reported FT-engineered PVA/PEG hydrogels with a 3D open porous structure exhibiting a relatively low compressive Young’s modulus of 0.069 MPa [15]. In contrast, Liu et al. demonstrated that engineering a concrete-like microstructure significantly enhances stiffness, achieving a compressive Young’s modulus of up to 2.5 MPa [14]. Inspired by cellular solids, the mechanical performance of such porous hydrogels can be further enhanced. In cellular solids, closed-cell domains contribute to load-bearing stiffness by resisting volumetric deformation, while open-cell regions facilitate fluid redistribution and energy dissipation [23,24]. This synergistic interplay between structural rigidity and fluid-mediated damping is critical for achieving both high strength and resilience in hydrated polymer networks, while also supporting fluid transport relevant to joint lubrication and nutrient exchange [25,26,27].

Although PVA/PEG hydrogels have been explored as candidate materials for artificial cartilage, the structure–property relationships governing their load-bearing and impact-resistant performance remain insufficiently understood [4,14]. In particular, native articular cartilage is routinely subjected to dynamic and impact loading (e.g., running and jumping), where its primary function is to dissipate mechanical energy, cushion impact, and distribute stress to protect subchondral bone [28,29]. However, most existing studies on PVA-based hydrogels focus on quasi-static mechanical properties, with limited investigation into rate-dependent mechanical behavior under physiologically relevant loading conditions. Unlike conventional freeze-thawed PVA-based hydrogels that generally exhibit relatively homogeneous open porous networks, a hybrid open–closed porous architecture may provide a more effective balance between stiffness and energy dissipation. In this design, closed-pore domains contribute to load-bearing resistance, whereas interconnected open-pore regions facilitate fluid redistribution and mechanical damping.

In this study, we develop a facile and scalable FT-engineered PVA/PEG hydrogel platform featuring a hybrid open–closed porous network. Notably, closed-pore domains are formed within the porous structure due to the entrapment of air bubbles in the highly viscous precursor solution, resulting in a combination of open and confined pore architectures. By systematically varying key parameters, including PVA molecular weight, polymer concentration, and the number of FT cycles, we establish clear correlations between network structure, water content, and mechanical performance. Beyond conventional quasi-static characterization, dynamic compression testing is employed to reveal strain-rate-dependent mechanical reinforcement, providing insight into hydrogel behavior under impact-like conditions. Furthermore, finite element simulations are conducted to elucidate the viscoelastic deformation and load-transfer mechanisms within the interconnected network, bridging experimental observations with underlying mechanics. Cytocompatibility and direct-contact cytotoxicity assays confirm the biological suitability of the hydrogels, supporting their potential application in cartilage tissue engineering.

## 2. Results and Discussion

**Figure 1:**
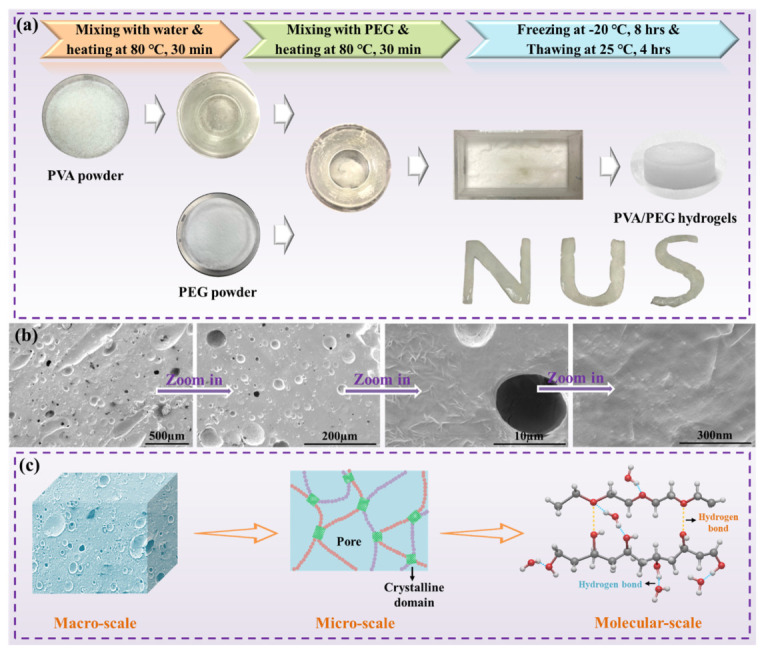
(**a**) Fabrication process, (**b**) morphological features at various scales (sample PPH2), and (**c**) multiscale design strategies of FT PVA/PEG hydrogels.

Figure 1a shows the facile fabrication process of PVA/PEG hydrogels, which can be readily shaped and molded into various geometries, demonstrating excellent formability and potential suitability for cartilage substitute applications. The estimated production cost of PVA/PEG hydrogels with 18 wt.% PVA and 18 wt.% PEG and subjected to 3 FT cycles is summarized in Table S1, accounting for raw materials, electricity, equipment, and labor. Owing to the simple and scalable fabrication process, the total production cost of the developed materials is approximately 0.11 USD per cm^3^, highlighting the cost-effectiveness and scalability of the proposed method. This processing philosophy is consistent with recent studies on lightweight porous materials and aerogels fabricated from low-cost or waste-derived resources, where simple solution processing and drying routes have been used to construct functional macroporous networks [30,31,32].

### 2.1. Morphology and Chemical Structure of FT Hydrogels

**Figure 2:**
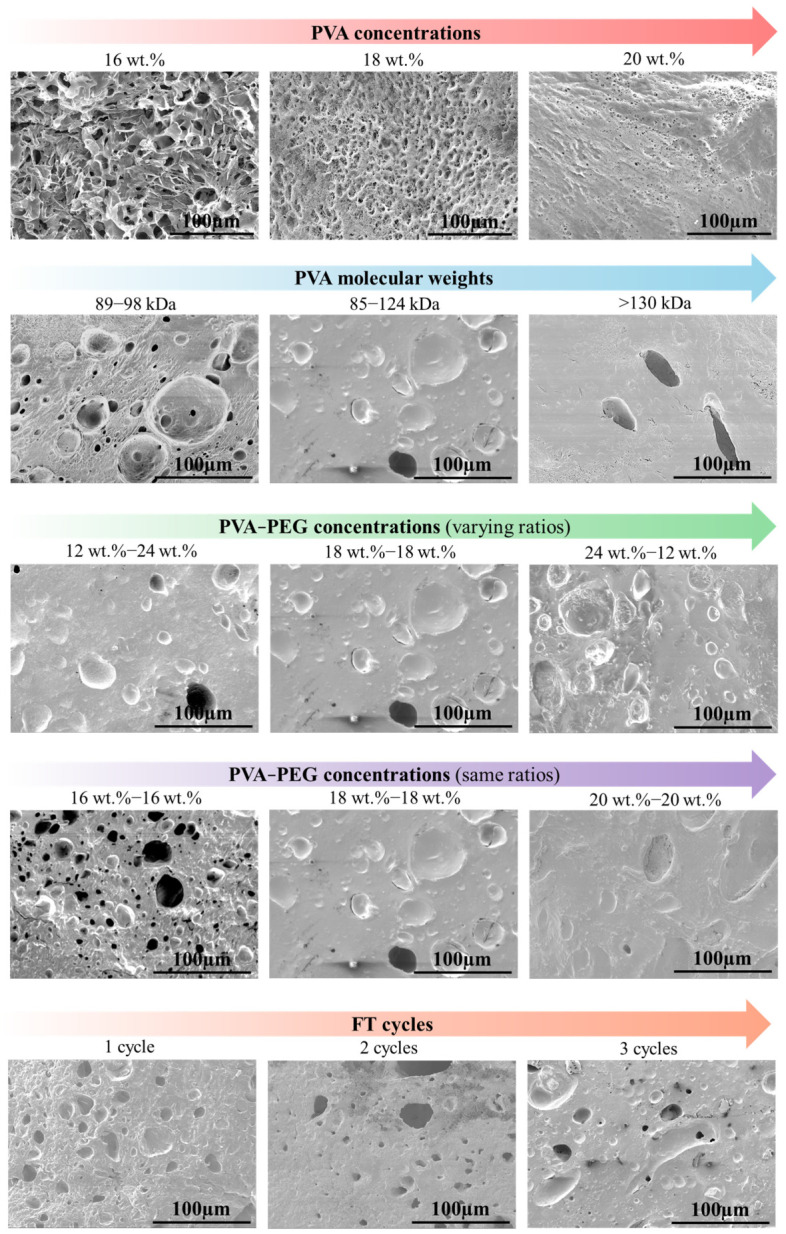
Morphologies of PVA hydrogels prepared with different PVA concentrations and morphological characteristics of PVA/PEG hydrogels fabricated using different PVA molecular-weights, PVA-PEG concentrations, and FT cycles.

Figure 2 presents morphologies of PVA hydrogels and PVA/PEG hydrogels with different specifications. In general, PVA hydrogels exhibit porous and homogeneous structures, characterized by macropores formed by melted ice crystals during FT cycles. Increasing the PVA concentration from 16 wt.% to 20 wt.% leads to a noticeably denser structure in PVA hydrogels, accompanied by a substantial reduction in pore size. This behavior can be attributed to the reduced water content and higher polymer concentration in the precursor solution, which restrict ice crystal growth during freezing and promote closer packing of PVA chains, thereby forming a more compact PVA-rich phase [12]. Unlike the PVA hydrogels’ structure, the incorporation of PEG results in a denser network structure featuring a combination of open (interconnected) pores and closed pores, as shown Figure 1b. This hybrid open–closed porous morphology distinguishes the developed PVA/PEG hydrogels from conventional homogeneous porous hydrogel networks and provides a structural basis for simultaneously improving load-bearing stiffness and energy dissipation [33]. The increased structural density of PVA/PEG hydrogels indicates a higher effective crosslinking density arising from enhanced intermolecular interactions between PVA and PEG chains [6]. Figure S1 further illustrates the morphological characteristics and porous microstructure of the PVA/PEG hydrogels (sample PPH2) at different magnifications. The closed-pore system exhibits a broad pore-size distribution ranging from approximately 2–3 µm to ~150 µm, which is associated with air entrapment introduced naturally during the stirring process. In contrast, the interconnected open-pore structures exhibit relatively smaller and more uniform pore sizes of approximately 1–10 µm. Furthermore, closed pores appear to dominate the hydrogel structure, with more than 90% of the observed pores on the surface exhibiting closed-pore characteristics.

Furthermore, PVA/PEG hydrogels fabricated using higher-molecular-weight PVA exhibit fewer pores, which are predominantly closed. This observation suggests that higher-molecular-weight PVA increases the viscosity of the PVA/PEG precursor solution, thereby promoting the entrapment of air bubbles during stirring. The elevated viscosity also restricts water mobility and distribution during mixing, leading to the formation of a denser polymer network with reduced pore interconnectivity after gelation [34]. At a constant total polymer concentration, increasing the PVA fraction further promotes structural densification and reduces open porosity. This behavior is attributed to the substantially higher molecular weight of PVA (85–124 kDa) compared to PEG (8 kDa), which increases solution viscosity and enhances air entrapment. Similar to pure PVA hydrogels, increasing the PVA-PEG concentration from 16 wt.%–16 wt.% to 20 wt.%–20 wt.% results in a markedly denser hydrogel structure with significantly fewer open pores.

In addition, a more compact PVA/PEG hydrogel structure is observed with increasing numbers of FT cycles. During the FT process, successive cycling drives polymer chains toward polymer-rich regions, where they progressively aggregate and crystallize into solid domains, as depicted in Figure 1c (middle) [12]. These crystalline regions act as rigid physical crosslinking points within the hydrogel network, further enhancing structural densification.

**Figure 3:**
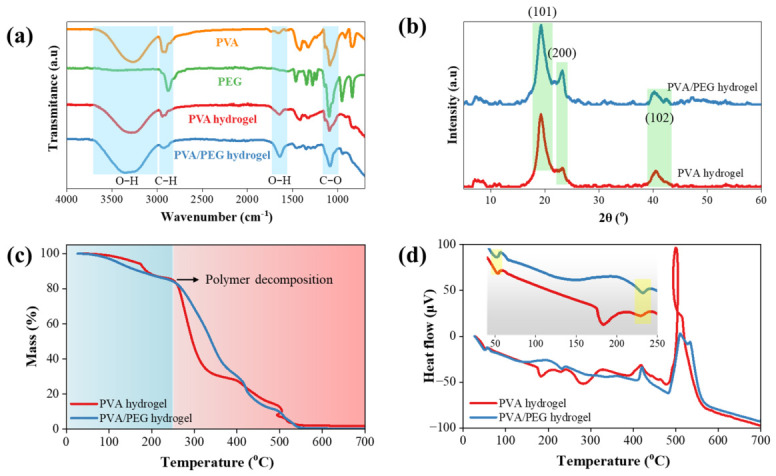
(**a**) FTIR spectra, (**b**) XRD patterns, (**c**) TGA and (**d**) DTA curves of PVA hydrogel (PH2), and PVA/PEG hydrogel (PPH2).

FTIR spectra of raw PVA and PEG, as well as the resulting PVA hydrogel (sample PH2) and PVA/PEG hydrogel (sample PPH2), are shown in Figure 3a. A broad band at approximately 3300 cm^−1^ is characteristic of O–H stretching, located adjacent to the C–H stretching peak at around 2900 cm^−1^ [35,36]. Peaks observed near 1650 cm^−1^ and 1090 cm^−1^ are attributed to O–H bending and C–O stretching, respectively [37]. In contrast to PVA, PEG does not exhibit characteristic O–H peaks but shows distinct C–O–C stretching peaks at around 1282 cm^−1^ and 1230 cm^−1^ [18,38]. Additional absorption bands appearing near 1420 cm^−1^, 1330 cm^−1^, 950 cm^−1^, and 840 cm^−1^ in both PVA and PEG correspond to –CH_2_– bending, –CH_2_– wagging, –CH_2_– rocking, C–C stretching, respectively [35,39,40,41]. Compared to raw PVA, both PVA and PVA/PEG hydrogels exhibit noticeable broadening and slight shifts of the O–H stretching band, indicating enhanced hydrogen bonding interactions among PVA chains, PEG chains, and water molecules, as visualized in Figure 1c (right). These interactions are further evidenced by the increased intensity of the O–H bending bands in both hydrogels, confirming the formation of an interconnected hydrogen-bonded network [6,42].

XRD is employed to characterize crystalline structures of PVA hydrogel (PH2) and PVA/PEG hydrogel (PPH2), and the corresponding diffraction patterns are displayed in Figure 3b. The XRD pattern of PVA hydrogel exhibits three characteristic diffraction peaks at 2θ = 19.1°, 23.1°, and 40.4°, which are assigned to (101), (200), and (102) crystalline planes of PVA, respectively [43,44]. Among these, the semi-crystalline (101) reflection is commonly associated with intermolecular ordering and hydrogen-bond-mediated interactions among PVA chains [45]. Both PVA and PVA/PEG hydrogels display a pronounced (101) peak, indicating the presence of strong hydrogen bonding among PVA chains within the hydrogel matrix. Notably, PVA/PEG hydrogel exhibits a sharper (200) diffraction peak compared with pristine PVA hydrogel, suggesting enhanced crystallinity or more ordered crystalline domains upon incorporation of PEG into the PVA network [14,46].

Figure 3c illustrates the thermal stability of PVA hydrogel (PH2) and PVA/PEG hydrogel (PPH2). As reported in the literature [35,47], both PVA and PEG begin thermal decomposition at around 250 °C, which is evident from the onset of major mass loss in the TGA curves. Below 250 °C, the observed weight loss is primarily attributed to the evaporation of physically bound and free water within the hydrogel network.

The thermal behaviors of hydrogels are further evaluated by the DTA curves in Figure 3d. Small endothermic transitions appearing near 50 °C in both PVA and PVA/PEG hydrogels are associated with the glass transition of PVA. Compared to neat PVA, which has the reported glass transition temperature of 82 °C [48], this decrease in the glass transition temperature is expected, as the presence of water and PEG plasticizes the PVA chains, increasing their mobility and flexibility under heating [49]. The broad endothermic region spanning from 50 °C to 200 °C in both PVA and PVA/PEG hydrogels corresponds to progressive water evaporation. Notably, this region is broader for PVA/PEG hydrogel, while a more distinct endothermic peak around 183 °C is observed for PVA hydrogel. This difference suggests stronger interactions between water molecules and the PVA-rich network compared to the PEG-modified system, indicating higher water-binding energy in PVA hydrogel [50]. The endothermic melting transitions of PVA and PVA/PEG hydrogels are observed at approximately 229 °C and 232 °C, respectively, demonstrating their high thermal tolerance. The slightly higher melting temperature of the PVA/PEG hydrogel further indicates an increased degree of crystallinity, which is consistent with the enhanced crystalline ordering revealed by the XRD analysis [4,48].

### 2.2. Mechanical Properties of FT Hydrogels

**Figure 4:**
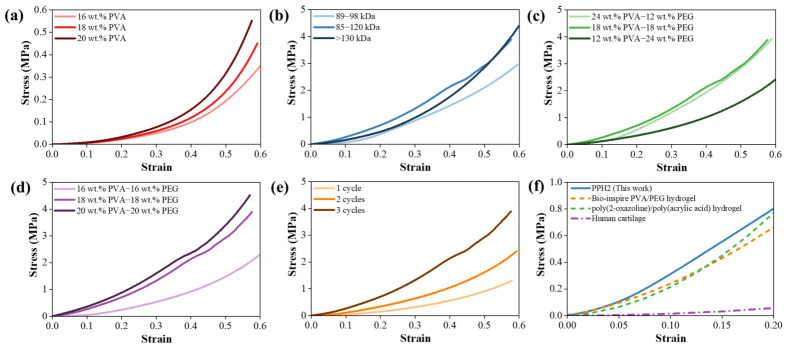
Compression stress–strain curves of (**a**) PVA hydrogels and PVA/PEG hydrogels with varying (**b**) PVA molecular weights, (**c**,**d**) PVA-PEG concentrations, and (**e**) the number of FT cycles. (**f**) A comparison in static compression stress–strain curves of PPH2, human cartilage [53] and other previously reported hydrogels [14,54].

Articular cartilage is a load-bearing tissue that primarily experiences compressive stresses, therefore, optimizing the compressive strength of hydrogel-based cartilage substitutes is essential [51]. The compressive properties of PVA and PVA/PEG hydrogels with varying PVA molecular weights, PVA and PEG concentrations, and numbers of FT cycles are comprehensively investigated, and the results are summarized in Figure 4. As shown in Figure 4a, the compressive Young’s modulus of pristine PVA hydrogels increases slightly from 0.08 MPa to 0.11 MPa as the PVA concentration increases from 16 wt.% to 20 wt.%. This enhancement is attributed to the formation of smaller and more compact pores at higher PVA concentrations, as discussed in the morphology, which reduces free volume and increases resistance to deformation under compressive loading. Nevertheless, the compressive Young’s modulus of PVA hydrogels remains below the reported range for native human articular cartilage (0.24–1.00 MPa) [52]. In contrast, incorporation of PEG as a physical crosslinker (18 wt.% PVA–18 wt.% PEG) results in a substantial increase in compressive Young’s modulus to 3.60 MPa, highlighting the effectiveness of PEG-induced physical crosslinking in reinforcing the PVA network and the significance of the pore structure in enhancing its load-bearing capability.

The influence of PVA molecular weight on the compressive strength of PVA/PEG hydrogels is shown in Figure 4b. In principle, increasing PVA molecular weight is expected to enhance compressive strength due to increased chain entanglement and a higher density of hydrogen bonds per chain [12]. However, the experimental results reveal a nonmonotonic trend. The compressive Young’s modulus increases from 1.16 MPa to 3.60 MPa as the PVA molecular weight increases from 89–98 kDa to 85–120 kDa but subsequently decreases to 1.77 MPa when the PVA molecular weight exceeds 130 kDa. A similar trend is observed for PVA/PEG hydrogels with varying PVA-to-PEG ratios (Figure 4c). The compressive Young’s modulus increases from 1.45 MPa for hydrogels containing 12 wt.% PVA–24 wt.% PEG to a maximum of 3.60 MPa for those with 18 wt.% PVA–18 wt.% PEG and then decreases to 1.35 MPa for 24 wt.% PVA–12 wt.% PEG hydrogels. These results suggest that PVA with moderate molecular weight (85–120 kDa) provides an optimal structural framework, while PEG acts as a flexible segment that facilitates physical crosslinking and network stabilization. Consequently, an optimal PVA-to-PEG mass ratio of 1:1 is identified for maximizing compressive performance. At this optimal ratio, the compressive Young’s modulus increases markedly from 0.55 MPa to 3.73 MPa as the polymer concentrations rise from 16 wt.%–16 wt.% to 20 wt.%–20 wt.% (Figure 4d). However, this enhancement becomes less pronounced when the concentration increases from 18 wt.%–18 wt.% to 20 wt.%–20 wt.%.

These trends also indicate that Young’s modulus exhibits a dependence on pore structure, initially increasing and subsequently decreasing with increasing closed porosity. Therefore, the hybrid open–closed porous architecture provides a structural balance in which closed pores contribute to load-bearing stiffness, while interconnected open pores facilitate water redistribution, local deformation, and energy dissipation during compression [55]. As a result, an optimal pore architecture, balancing closed and open porosity, is required to maximize stiffness.

The effect of FT cycling on the compression strength of PVA/PEG hydrogels is depicted in Figure 4e. The compressive Young’s modulus increases steadily from 0.27 MPa after one FT cycle to 3.60 MPa after three cycles, which can be attributed to progressive crystallization during repeated FT processing, leading to the formation of a stronger physical crosslinking network [56]. Nevertheless, to balance mechanical performance with fabrication efficiency and practical applicability, the number of FT cycles is fixed at three for subsequent experiments.

Compared with native human articular cartilage, our developed PVA/PEG hydrogel (PPH2) exhibits a markedly higher Young’s modulus, 3.60 ± 0.67 MPa versus 0.42 MPa [11,53]. When benchmarked against recently reported hydrogel-based artificial cartilage systems (Figure 4f), the PVA/PEG hydrogel also demonstrates superior compressive performance. These results highlight the strong potential of the developed PVA/PEG hydrogel as a load-bearing cartilage substitute from a mechanical strength perspective.

**Figure 5:**
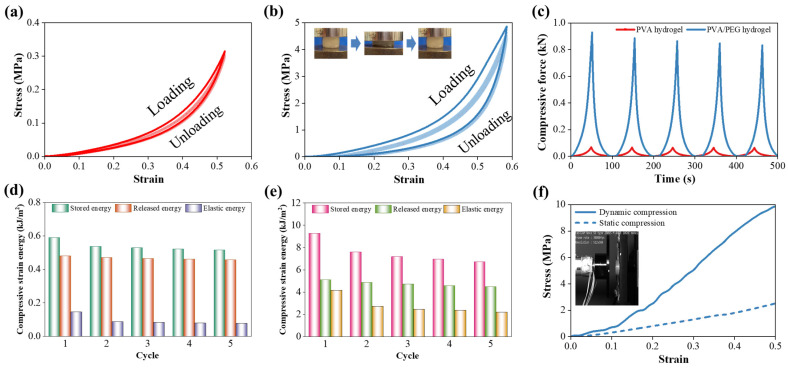
(**a**,**b**) Loading–unloading compression and (**c**) time evolution of compressive force of PVA (PH2) and PVA/PEG (PPH2) hydrogels. Compression strain energy of (**d**) PVA hydrogel (PH2) and (**e**) PVA/PEG (PPH2) hydrogel. (**f**) Dynamic compression stress–strain response of PVA/PEG hydrogel (PPH2).

The dynamic stability of hydrogels, which requires resilience under repeated mechanical loading, is a critical consideration for their load-bearing cartilage applications. The loading–unloading stress–strain responses of PVA and PVA/PEG hydrogels over five consecutive cycles are presented in Figure 5a,b to describe their energy dissipation mechanisms. Both PVA and PVA/PEG hydrogels exhibit highly resilient compressive behavior under cyclic compression (Figure 5c). Notably, PVA/PEG hydrogel displays a pronounced hysteresis loop compared to PVA hydrogel, indicating a substantially enhanced energy-dissipation capability. This behavior is attributed to the higher crosslinking density in the PVA/PEG network as well as its partially interconnected open-pore structure, which enables more efficient dissipation of external mechanical energy [6,57]. This can also be observed in the much higher elastic energy (area of hysteresis loops) of PVA/PEG hydrogel (2.21–4.17 kJ/m^2^ versus 0.06–0.11 kJ/m^2^) (Figure 5d,e). Although the stored energy of PVA/PEG hydrogel (total area under unloading curves) decreases faster than PVA hydrogel in the first loading cycle due to rupture of the rich hydrogen-bond network, both almost remain their stored energy and released energy (total area under unloading curves) from the second loading–unloading cycle onward. This cyclic hysteresis behavior suggests that the PVA/PEG hydrogel dissipates mechanical energy through a viscoelastic response, rather than functioning solely as a stiff load-bearing material. This behavior is relevant to native cartilage, where energy dissipation under compression is associated with time-dependent deformation of the hydrated porous matrix and redistribution of interstitial fluid [58]. Although the PVA/PEG hydrogel exhibited promising resilience under cyclic loading, long-term fatigue resistance and wear behavior under repeated physiological loading and lubrication conditions should be further evaluated in future studies to fully assess its durability as a cartilage substitute [59,60].

Articular cartilage in synovial joints is routinely subjected to dynamic and impact loading during activities such as running and jumping. A primary function of articular cartilage is therefore to absorb impact energy, providing cushioning and distributing loads to prevent stress concentration in the underlying subchondral bone [11]. Response of PVA/PEG hydrogel (PPH2) subjected to direct impact by the striker at 2 m/s is demonstrated in Figure 5f and Video S1. In human articular cartilage, the strain rates generated during daily movements are typically in the range of 10–100 s^−1^, whereas traumatic events such as traffic accidents can generate strain rates approaching 1000 s^−1^ [11]. To further challenge the mechanical robustness of the developed hydrogel, dynamic compression testing is conducted at a loading velocity of 2 m/s, corresponding to a strain rate of approximately 267 s^−1^. Consistent with the quasi-static compression results, the stress–strain curves under dynamic loading exhibit similar elastic behavior. However, the dynamic compressive Young’s modulus is nearly twice that obtained under quasi-static conditions. At a compressive strain of 0.5, the dynamic compressive stress reaches approximately 10 MPa, which is about five times higher than that measured under quasi-static loading. This pronounced strain-rate-dependent stiffening behavior originates from the coupled viscoelastic and poroelastic nature of the hydrogel network, similar to that observed in soft biological connective tissues [61]. At lower loading rates, polymer chains have sufficient time to rearrange and relax, thereby accommodating applied deformation and resulting in lower compressive stress [62]. Additionally, water molecules confined within the hydrogel network can migrate through interconnected pores during slow compression, thereby reducing internal hydraulic resistance [63]. In contrast, under high-rate dynamic loading, both polymer-chain relaxation and water transport become kinetically restricted. The limited mobility of polymer chains suppresses structural relaxation, while trapped water within the hydrogel network generates transient hydraulic pressure that resists rapid volumetric deformation [61,62,63]. Consequently, the hydrogel exhibits a significantly stiffer mechanical response and enhanced energy dissipation capability under impact loading conditions. These results demonstrate that PVA/PEG hydrogel exhibits effective impact-absorption capability and rate-dependent stiffening, indicating a viscoelastic load-dissipation response that is relevant to cartilage substitutes subjected to frequent dynamic impact loading.

**Figure 6:**
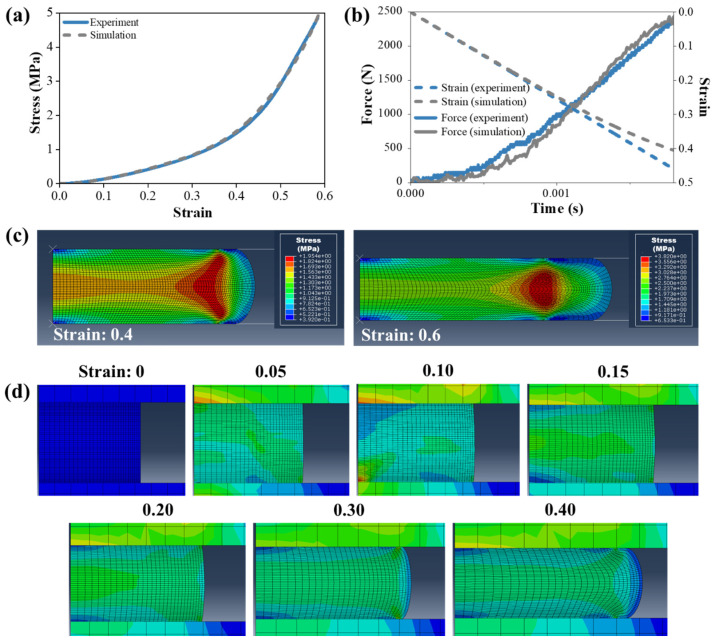
Comparisons between FE simulations and experimental results for PVA/PEG hydrogel (PPH2) under (**a**) quasi-static compression and (**b**) dynamic compression. FE-predicted stress distribution and specimen deformation profiles of PVA/PEG hydrogel (PPH2) under (**c**) quasi-static compression and (**d**) dynamic compression with a striker impact velocity of 2.0 m/s.

Finite element (FE) simulations are performed using ABAQUS Explicit. An axisymmetric FE model is constructed to simulate the quasi-static compression of the PVA/PEG hydrogel specimen. The cylindrical specimen, with an aspect ratio of 1, is placed between two rigid steel plates and discretized using CAX4R rectangular axisymmetric elements. Geometric nonlinearity is enabled throughout the simulations to account for the large deformation behavior of the hydrogel during compression. The steel plates are modelled as linear-elastic materials with a Young’s modulus of 200 GPa and a Poisson’s ratio of 0.3. The hydrogel specimen is described using a hyper-elastic model based on the Marlow strain energy potential, calibrated directly from the experimentally measured uniaxial compression stress–strain response [11]. A Poisson’s ratio of 0.47 is adopted for the hydrogel material to represent its nearly incompressible behavior. The lower rigid plate is fully constrained, while a prescribed vertical displacement is applied to the upper rigid plate to simulate the compression process. Hard normal contact and frictional tangential contact are defined between the hydrogel specimen and the rigid plates, with a friction coefficient of 0.1, in order to reproduce the experimentally observed barreling deformation behavior. The stress–strain response predicted by the FE simulation shows excellent agreement with experimental data (Figure 6a), validating the capability of the FE model to accurately capture the mechanical behavior of PVA/PEG hydrogel under quasi-static compression. In addition, the simulated deformation profile agrees well with the experimentally observed specimen barreling during compression.

Similarly, an axisymmetric FE model is established to simulate the direct-impact Hopkinson bar test on the PVA/PEG hydrogel specimen. In this FE model, the specimen is positioned between the striker flange and a steel support plate, both discretized using CAX4R axisymmetric elements. The steel plate and striker flange are modeled as linear-elastic materials with a Young’s modulus of 200 GPa, Poisson’s ratio of 0.3, and density of 7800 kg/m^3^, while the aluminum alloy striker is assigned a Young’s modulus of 70 GPa, Poisson’s ratio of 0.33, and density of 2700 kg/m^3^. The hydrogel specimen is described using a Marlow hyper-elastic constitutive model, calibrated from the experimentally measured dynamic compression stress–strain response [11]. The simulated stress–strain response also exhibits excellent agreement with experimental results (Figure 6b), confirming that the FE model accurately captures the rate-dependent mechanical behavior of PVA/PEG hydrogel under high-strain-rate compression.

The stress distribution within PVA/PEG hydrogel under quasi-static compression at nominal strains of 0.4 and 0.6 is shown in Figure 6c. A non-uniform stress field is observed, with pronounced stress localization near the specimen-platen contact edges due to constrained lateral deformation. The stress magnitude gradually decreases along the specimen length, indicating effective load transfer through the interconnected hydrogel network. At higher compressive strain, enhanced lateral expansion leads to an increase in peak stress and a redistribution of the stress field, contributing to the observed nonlinear mechanical response of the hydrogel. Figure 6d illustrates the temporal evolution of internal stress during dynamic compression. The simulations reveal a clear time-dependent stress propagation, where stress waves initiate at the loading surface and progressively transmit into the bulk material. Compared to quasi-static loading, dynamic compression results in a more distributed stress field, consistent with the viscoelastic nature of the PVA/PEG hydrogel and its strain-rate dependent mechanical behavior, which are important for reducing localized stress accumulation in cartilage-relevant load-bearing environments [64].

### 2.3. Water Content and Water Contact Angles of FT Hydrogels

**Figure 7:**
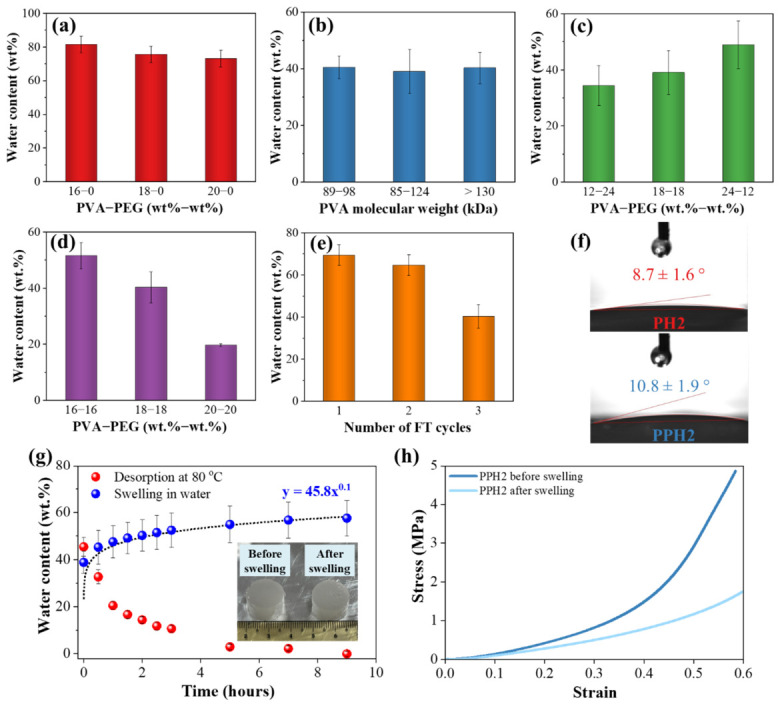
Water contents of (**a**) PVA hydrogels and PVA/PEG hydrogels with (**b**) varying PVA molecular weights, (**c**,**d**) PVA-PEG concentrations, and (**e**) the number of FT cycles. (**f**) Water contact angle of PVA (PH2) and PVA/PEG hydrogels (PPH2). (**g**) Water swelling and desorption (at 80 °C) properties of PVA/PEG hydrogel sample PPH2. (**h**) Compression stress–strain curves of PPH2 before and after swelling in water for 24 h.

Figure 7a–e summarizes the water contents of PVA and PVA/PEG hydrogels with different compositions. For pristine PVA hydrogels (Figure 7a), their water contents range from 73.2 wt.% to 81.6 wt.%, which in line with previously reported values for FT PVA hydrogels [2]. The water content decreases with increasing the PVA concentration (16–20 wt.%) because of the formation of a denser polymer network, as observed in Figure 2. Upon incorporation of PEG into the PVA network, the water content decreases markedly to 39.1 ± 7.8 wt.% for the PVA/PEG hydrogel (PPH2). This reduction is understandable, as the total polymer content in the PVA/PEG system is substantially higher, consisting of 18 wt.% PVA–18 wt.% PEG, resulting in a more compact network with reduced free volume for water remaining in the hydrogel network. Interestingly, PVA/PEG hydrogels prepared using different PVA molecular weights exhibit insignificant differences in water content (Figure 7b) despite their distinct morphological features.

For PVA/PEG hydrogels with varying PEG-to-PVA ratios at a fixed total polymer concentration, decreasing the PEG mass fraction leads to higher water content in hydrogels (Figure 7c). As shown in Figure 2, these samples exhibit similarly dense structures, even though increasing the PVA concentration results in denser networks. This illustrates the higher hydrophilicity of PVA compared to PEG, which could be due to the stronger hydrogen bonds of –OH groups with water molecules compared to C–O–C groups (Figure 1c, right) [48,65]. This is further supported by the lower water contact angle of PVA hydrogel (PH2) than that of PVA/PEG hydrogel (PPH2) in Figure 7f.

Similar to pristine PVA hydrogels, increasing the total concentration of PVA and PEG markedly reduces the water content of PVA/PEG hydrogels (Figure 7d). For instance, hydrogels containing 20 wt.% PVA–20 wt.% PEG exhibit a water content as low as 19.7 ± 0.4 wt.%. This downward trend is attributed to the formation of a more compact polymer network at higher total polymer concentrations, as evidenced by the denser structures shown in Figure 2. The influence of FT cycles on the water content of PVA/PEG hydrogels is presented in Figure 7e. The water content decreases with increasing FT cycles, with a more pronounced reduction observed after the second cycle. A greater fraction of PVA and PEG chains is driven into polymer-rich regions and crystallizes into the solid phase with the increasing number of FT cycles, thereby increasing network density [12]. This effect is further corroborated by the increasingly compact structures observed in PVA/PEG hydrogels with higher numbers of FT cycles (Figure 2).

In comparison with the trends observed in Figure 4, the compressive Young’s modulus increases as the water content decreases with increasing polymer concentrations and increasing number of FT cycles. However, this trend does not necessarily imply that mechanical behavior is governed solely by water content. Instead, enhanced mechanical performance is more likely associated with the increased crystallized solid phase and densification of the hydrogel network [66]. Furthermore, at the same total concentration of PVA and PEG, the compressive Young’s modulus does not exhibit a direct correlation with the water content of the hydrogels (Figure 4b,c). Rather, the mechanical properties appear to be governed predominantly by the internal network structure and morphological characteristics of the hydrogels.

In addition, the hydrophilicity of hydrogels is closely associated with their lubrication performance and the transport of synovial fluid for nutrient exchange in the joints [46]. As illustrated in Figure 7f, both PVA and PVA/PEG hydrogels exhibit strong hydrophilicity, with static water contact angles of 8.7 ± 1.6° and 10.8 ± 1.9°, respectively. Such high surface wettability is expected to facilitate the formation of a stable hydration layer at the hydrogel surface, which is important for reducing interfacial friction during articulation and supporting fluid transport within the polymer network. This hydration-mediated lubrication mechanism is relevant to native cartilage, where low friction is largely maintained by the hydrated surface and synovial fluid at the articulating interface [67,68]. However, direct measurements of friction coefficient and wear behavior under physiologically relevant lubrication and articulating conditions should be conducted in future studies to further evaluate the tribological performance of the PVA/PEG hydrogel as a cartilage substitute.

The water retention behavior of PVA/PEG hydrogel (PPH2) is further investigated through water desorption tests conducted at 80 °C and swelling experiments in DI water, as shown in Figure 7g. The desorption temperature of 80 °C is selected to accelerate water evaporation and enable clear evaluation of the water-binding strength within the hydrogel network while preserving its structural integrity. During water desorption at 80 °C, approximately 70 wt.% of the water is removed within the first 2 h, which is associated with loosely bound water located at the hydrogel surface or within the pore spaces. Complete water removal from PVA/PEG hydrogel is achieved after approximately 9 h of drying at 80 °C. In contrast, during water uptake experiments, PVA/PEG hydrogel gradually absorbs water and reaches an equilibrium water content of 63.5 ± 6.7 wt.% within 24 h, falling within the range of water content in joint cartilage (60–85 wt.%) [46]. The swelling kinetics nearly follow a zero-order model, indicating a constant swelling rate that is independent of the water amount. This behavior suggests that water uptake is predominantly governed by steady diffusion through a uniformly hydrated polymer network rather than by polymer chain relaxation to create additional available sites [69]. After reaching equilibrium swelling, a slight increase in sample dimensions is observed (inset of Figure 7g). This leads to a modest reduction in mechanical strength. As shown in Figure 7h, the compressive Young’s modulus of PVA/PEG hydrogels decreases from 2.7 MPa to 1.7 MPa as the water content increases from 39.1 ± 7.8 wt.% to 63.5 ± 6.7 wt.% upon the swelling test.

### 2.4. Biocompatibility of FT Hydrogels

**Figure 8:**
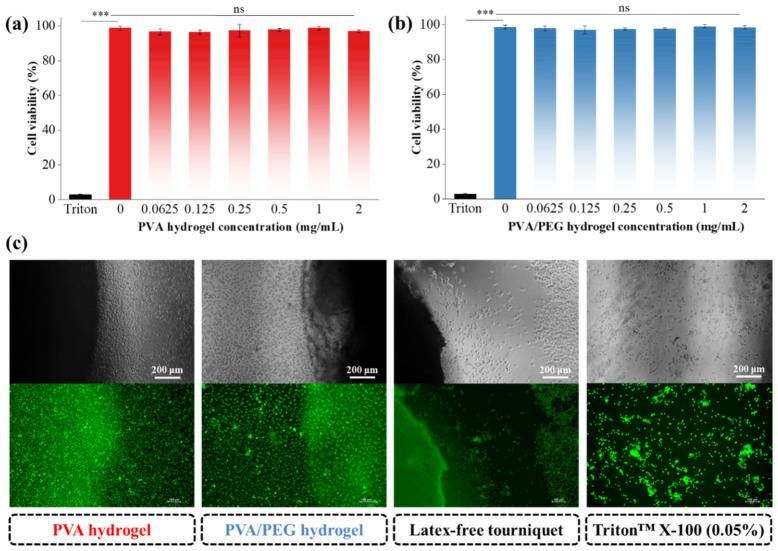
In vitro cytotoxicity assessment of (**a**) PVA hydrogel (PH2) and (**b**) PVA/PEG hydrogel (PPH2) in L929 fibroblasts after 48 h exposure using the MTT assay. Statistical significance is denoted as ns (not significant), *** *p* < 0.001. (**c**) Direct contact cytotoxicity evaluation of PVA and PVA/PEG hydrogels versus a latex-free tourniquet and Triton™ X-100 (0.05%) on L929 fibroblasts visualized by SYTO™ 9 nucleic acid staining. Scale bars = 200 μm (**top**) and 100 μm (**bottom**).

In vitro cytotoxicity of PVA and PVA/PEG hydrogels is evaluated using the MTT assay, as shown in Figure 8. Untreated L929 fibroblasts (0 mg/mL) exhibit high metabolic activity (98.62 ± 1.07%). Exposure to PVA hydrogel extracts over a concentration range of 0.0625–2 mg/mL resulted in cell viabilities comparable to the untreated control (96.38–98.71%) (Figure 8a). A similar trend is observed for the PVA/PEG hydrogel (Figure 8b), with the cell viability remaining near baseline (97.00–98.97%) across all tested extract concentrations. Although the Kruskal–Wallis test indicates a significant overall difference among groups (*p* = 0.000269 for PVA hydrogel and *p* = 0.000675 for PVA/PEG hydrogel test), this effect is driven exclusively by the positive control, Triton™ X-100 (0.05%). Specifically, Triton™ X-100 caused a pronounced reduction in cell viability (2.73 ± 0.43%), which is significantly different from the untreated control (Dunn-adjusted *p* = 0.000698 and 0.00188, ***, for PVA hydrogel and PVA/PEG hydrogel tests, respectively). Together, these results confirm that neither PVA nor PVA/PEG hydrogels exhibit measurable cytotoxicity under the conditions tested, supporting their cytocompatibility for tissue engineering applications.

The potential cytotoxic effects of PVA hydrogel and PVA/PEG hydrogel are further evaluated using a direct contact cytotoxicity assay, as illustrated in Figure 8c. For both PVA hydrogel and PVA/PEG hydrogel, L929 fibroblasts remain viable and uniformly distribute around and beneath the material disks after 48 h of direct contact. No cell-free zones, concentric rings of dead cells, or reductions in SYTO™ 9 fluorescence intensity are observed in the vicinity of either material. These findings indicate that neither material releases cytotoxic leachable nor induces contact-dependent cytotoxicity under the experimental conditions. In contrast, the latex-free tourniquet positive control produced a pronounced, well-defined cell-free zone surrounding the material. SYTO™ 9 fluorescence is absent in the immediate contact region, while viable cells are observed at greater distances from the tourniquet, confirming a localized cytotoxic response characteristic of contact-mediated toxicity. As expected, treatment with Triton™ X-100 (0.05%) results in a near-complete loss of cellular integrity across the entire well. Since SYTO™ 9 stains cellular nucleic acids and does not strictly discriminate between live and dead cells [70], the intense fluorescence observed in Triton-treated samples is attributed to membrane disruption that facilitates dye penetration and binding to exposed nucleic acids, despite the loss of cell viability.

Taken together, these results demonstrate that both PVA and PVA/PEG hydrogels exhibit no detectable cytotoxicity in the direct-contact assay, as evidenced by the absence of localized or global cytotoxic effects. This observation complements the MTT viability results and further supports the cytocompatibility of these materials for tissue engineering applications, where direct interaction with living cells is unavoidable. Further chondrocyte-specific assays, including adhesion, proliferation, and extracellular matrix production, should be conducted in future studies to better assess the translational relevance of the PVA/PEG hydrogel as a cartilage substitute.

## 3. Conclusions

In this work, load-bearing PVA/PEG hydrogels were successfully developed using a facile, scalable, and cost-effective FT method for potential artificial cartilage replacement. The effects of polymer composition and processing parameters on water content and mechanical performance were systematically evaluated, leading to an optimized formulation consisting of 18 wt.% PVA (85–124 kDa) and 18 wt.% PEG subjected to three FT cycles. The resulting hydrogels exhibit a hybrid porous architecture with interconnected open and closed macropores, high water content (39.1 ± 7.8 wt.%), and excellent resilience under repeated compressive loading. Notably, the optimized PVA/PEG hydrogels with balanced open–closed porosity achieve an exceptional static compressive Young’s modulus of 3.60 ± 0.67 MPa, approximately nine times higher than that of native human cartilage and superior to most previously reported hydrogel-based cartilage substitutes. Under dynamic compression at a loading rate of 2 m/s, the hydrogels display nearly double the compressive modulus compared to static conditions, highlighting their strong potential to withstand impact loading associated with physiological activities such as running and jumping. FE simulations further confirm efficient load transfer throughout the interconnected hydrogel network. In vitro cytotoxicity assessments and direct contact cytotoxicity assays demonstrate excellent cytocompatibility, supporting the suitability of these materials for tissue engineering applications. Overall, the combination of high water content, outstanding static and dynamic mechanical performance, structural integrity, and cytocompatibility positions the developed PVA/PEG hydrogels as promising candidates for load-bearing cartilage replacement. Looking ahead, broader benchmarking against native cartilage and previously reported hydrogel systems should be conducted, including long-term fatigue resistance, wear behavior, frictional performance, and stability/bioactive behavior under physiologically relevant loading, lubrication, and simulated body-fluid conditions.

## 4. Materials and Methods

### 4.1. Materials

Fully hydrolyzed polyvinyl alcohol (PVA) with different molecular weight ranges (89–98 kDa, 85–124 kDa, and >130 kDa) was purchased from Merck, Sigma Aldrich Pte Ltd. (Singapore). Polyethylene glycol (PEG) with an average molecular weight of 8000 Da was supplied from Merck, Sigma Aldrich Pte Ltd. (Singapore). The materials used for cell viability experiments included Dulbecco’s Modified Eagle’s Medium (DMEM)/F12 Ham 3:1 mixture (Himedia, Thane, India), fetal bovine serum (Sigma-Aldrich, St. Louis, MO, USA), 1X antibiotics (Bio Basic, Markham, ON, Canada), Triton™ X-100 (Bio Basic, Markham, ON, Canada), dimethyl sulfoxide (Sigma-Aldrich, St. Louis, MO, USA), and L929 mouse fibroblast cells (ATCC: CCL-1). A latex-free tourniquet (Becton Dickinson, Franklin Lakes, NJ, USA) and SYTO™ 9 nucleic acid stain (Thermo Fisher Scientific, Waltham, MA, USA) were employed in direct contact tests. All chemicals were used as received, and all solutions were prepared with deionized water.

### 4.2. Fabrication of PVA/PEG Hydrogels

The fabrication process of FT PVA/PEG hydrogels is depicted in Figure 1. Briefly, PVA powder is dissolved in deionized water at 80 °C under stirring at 300 rpm for 30 min to obtain a transparent PVA solution. Subsequently, PEG powder is added to the PVA solution, and the mixture is further stirred at 80 °C for 30 min to ensure complete dissolution of PEG. At this stage, a gel-like phase is separated from the solution. The resulting mixture, containing both the gel and liquid phases, is then poured into molds with gentle compression applied to the gel to ensure complete filling and intimate contact with the mold surfaces. The sample is allowed to rest at room temperature for 4 h to prevent thermal shock prior to the FT cycling process. Each FT cycle consists of freezing at −20 °C for 8 h in a refrigerator followed by thawing at room temperature for 4 h. The compositions and processing parameters of PVA/PEG hydrogels prepared in this study are summarized in Table 1. PVA with different molecular weight ranges (89–98 kDa, 85–124 kDa, and >130 kDa) is investigated. The compositions of PVA and PEG in the hydrogels vary from 12 wt.% to 24 wt.% relative to the total mass of the mixture (PVA, PEG, and water). In addition, the influence of the number of FT cycles is evaluated over a range of 1–3 cycles.

### 4.3. Characterization

Morphologies of hydrogels are characterized by a field emission scanning electron microscope (FE-SEM, Hitachi S4300, Hitachi Ltd., Tokyo, Japan). Prior to observation, hydrogel samples are freeze-dried under vacuum for 48 h to remove water and then sputter-coated with a thin gold layer. Chemical structures of raw materials and hydrogels are analyzed by Fourier transform infrared spectroscopy (FTIR, Bruker VERTEX 80v, Bruker Optics, Ettlingen, Germany), scanning from 4000 to 400 cm^−1^ at a resolution of 1 cm^−1^. Crystalline structures of hydrogels are confirmed by X-ray diffraction (XRD, Shimadzu XRD-6000, Shimadzu Corporation, Kyoto, Japan) using Cu-K_α_ radiation source (λ = 0.1506 nm) with a scanning range of 5–60° and a scan rate of 2°/min. Surface hydrophilicity of hydrogels is assessed via static water contact angle measurements using a video contact angle goniometer (VCA Optima, AST Products Inc., Inc., Billerica, MA, USA). A 5 μL deionized water droplet is deposited onto the sample surface using a syringe, and the contact angle is determined by an image-analysis software. Measurements are performed at multiple locations on each sample, and the reported values represent the average. All FE-SEM, FTIR, XRD, and water contact angle measurements are conducted at room temperature. Thermal stability of hydrogels is investigated by thermogravimetric analysis (TGA) and differential thermal analysis (DTA) using a thermogravimetric analyzer (Shimadzu DTG-60H). Samples are heated from 25 °C to 700 °C in air at a constant heating rate of 10 °C/min.

Static compression properties of hydrogels are evaluated using a load frame machine (Series 5500, Instron, Norwood, MA, USA) in accordance with the ASTM C165 standard. Compressive Young’s modulus is determined as the slope coefficient in the linear region of the corresponding stress–strain curves. For each specification, more than three samples are tested to obtain the representative results. Dynamic compression tests are conducted using a homemade Hopkinson bar device, as detailed in our previous study [71]. An aluminum striker with a flat-end flange is launched at a velocity of 2 m/s by a gas gun to impinge the hydrogel specimen directly, which is mounted on a circular back plate. Both static and dynamic compression tests are performed under ambient room conditions using cylindrical hydrogel samples with dimensions of approximately 20 mm in diameter and 7–8 mm in thickness.

Water content of hydrogels is determined using Equation (1) based on their initial mass ($m_{wet}$) and final mass ($m_{dry}$) after drying at 80 °C overnight.

(1) $$Water\;content\left(wt.\%\right)=\frac{m_{wet}-m_{dry}}{m_{wet}}\times 100\%$$

Water desorption behaviors of hydrogels are evaluated by monitoring the decrease in their water content as a function of drying time at 80 °C. Swelling properties are examined by immersing wet hydrogel samples into deionized water under ambient conditions and measuring the evolution of water content over time.

The in vitro cytotoxicity of PVA and PVA/PEG hydrogel is evaluated using an MTT assay on L929 mouse fibroblast cells (ATCC: CCL-1), in accordance with established protocols [72,73]. Briefly, each material is sterilized, accurately weighed, and extracted by immersion in complete cell culture medium (DMEM/F12, 3:1 ratio, supplemented with 10% fetal bovine serum and 1X antibiotics) for 48 h at 37 °C. The resulting extraction solutions are sterilized by filtration through a 0.22 µm membrane filter. L929 cells are seeded into 96-well plates at a density of 5 × 10^3^ cells per well and allowed to attach for 24 h. The culture medium is then replaced with 100 µL of fresh medium containing the material extracts at concentrations of 2, 1, 0.5, 0.25, 0.125, 0.0625, or 0 mg/mL (negative control). A solution containing 0.05% (*v*/*v*) Triton™ X-100 serves as the positive cytotoxic control. Cells are incubated with the extracts for 48 h at 37 °C in a humidified atmosphere containing 5% CO_2_. Following exposure, MTT solution (5 mg/mL) is added to each well at 10% (*v*/*v*) and incubated for 3 h to allow formation of formazan crystals. The medium is subsequently removed, and 100 µL of dimethyl sulfoxide is added to dissolve the formazan product. Absorbance is measured at 570 nm using a Multiskan Ascent microplate reader (354-02133, Thermo Fisher Scientific, Waltham, MA, USA). Cell visualizations are conducted using an Eclipse Ti2-U inverted microscope (Nikon, New York, NY, USA). Cell viability is calculated according to Equation (2):

(2) $$Cell\;viability\left(\%\right)=\frac{{OD}^{\prime }}{OD}\times 100\%$$

where OD′ and OD represent the optical density of treated cells and untreated control cells, respectively.

All data are presented as mean ± standard deviation and analyzed using RStudio version 2024.12.0+467. Overall differences among experimental groups are assessed using the nonparametric Kruskal–Wallis test. When statistically significant differences are detected, Dunn’s post hoc multiple comparisons test is performed to compare each treatment group with the untreated control (0 mg/mL), with *p*-values adjusted using the Holm correction method. A *p*-value < 0.05 is considered statistically significant.

Direct contact cytotoxicity assays enable the detection of toxic leachable as well as localized cytotoxic responses associated with PVA and PVA/PEG hydrogels. This assay is based on the principle that materials releasing harmful substances or exhibiting surface-associated toxicity will induce cell death in directly contacting or adjacent cells, resulting in localized cell-free zones or a broader loss of cellular viability [74,75,76]. L929 mouse fibroblasts are seeded into 48-well culture plates at a density of 1.0 × 10^4^ cells per well and cultured for 24 h to allow stable cell attachment and spreading. After cell adhesion, disk-shaped samples of PVA hydrogel or PVA-PEG hydrogel (3 mm diameter, 1 mm thickness) are gently placed onto the cell monolayer at the center of each well. The selected dimensions ensured sufficient material-cell contact while avoiding excessive mechanical compression of the underlying cells, consistent with commonly applied solid-material direct contact cytotoxicity assays. Two positive controls are included to validate assay sensitivity. A latex-free tourniquet is used as a localized cytotoxic positive control, since elastomeric materials are known to induce contact-dependent cytotoxic responses, typically manifested as a surrounding cell-free zone. In addition, Triton™ X-100 (0.05%) is used as a soluble positive control to induce global cell death across the entire well. Cells cultured without any materials serve as the negative control. Following placement of the materials, cells are cultured for an additional 48 h. Subsequently, cell viability is assessed using SYTO™ 9 green, fluorescent nucleic acid stain. Cells are gently washed with phosphate-buffered saline (PBS) and incubated with SYTO™ 9 solution prepared according to the manufacturer’s instructions for 15 min at room temperature in the dark. Cells are then washed to remove excess dye and immediately imaged using a fluorescence microscope. SYTO™ 9 stains nucleic acids of cells with intact membranes. This staining is used for qualitative visualization of viable cell distribution and detection of localized cytotoxic effects, rather than quantitative assessment of cell viability.

## Figures and Tables

**Table 1:** Specifications of PVA/PEG hydrogels developed by the FT method.

Sample	PVA Molecular Weight (kDa)	PVA Concentration (wt.%)	PEG Concentration (wt.%)	Number of FT Cycles
PH1	85–124	16	0	3
PH2	85–124	18	0	3
PH3	85–124	20	0	3
PPH1	89–98	18	18	3
PPH2	85–124	18	18	3
PPH3	>130	18	18	3
PPH4	85–124	12	24	3
PPH5	85–124	24	12	3
PPH6	85–124	16	16	3
PPH7	85–124	20	20	3
PPH8	85–124	18	18	2
PPH9	85–124	18	18	1

## Data Availability

The raw data supporting the conclusions of this article will be made available by the authors on request.

## Supplementary Materials

The following supporting information can be downloaded at: https://www.mdpi.com/article/10.3390/gels12060494/s1, Table S1: Estimated production cost of PVA/PEG hydrogels; Figure S1: SEM images showing morphological characteristics and porous microstructure of PVA/PEG hydrogels (PPH2) at different magnifications; Video S1: Dynamic compression of PVA/PEG hydrogel (PPH2) at the loading rate of 2 m/s.

## Abbreviations

The following abbreviations are used in this manuscript:

PVA	Polyvinyl alcohol
PEG	Polyethylene glycol
FT	Freeze-thaw
3D	Three dimensional
FTIR	Fourier transform infrared spectroscopy
XRD	X-ray diffraction
TGA	Thermogravimetric analysis
DTA	Differential thermal analysis
FE	Finite element
SEM	Scanning electron microscopy
