# Supplementary material for: Freeze–Thaw-Induced Hybrid Porous PVA/PEG Hydrogels with Dynamic Load-Dissipation Capability for Cartilage Substitutes
Source: Gels. 2026 Jun 2;12(6):494. doi: 10.3390/gels12060494 (PMC13298136; doi:10.3390/gels12060494)
Supplement: Supplementary file 1 [file gels-12-00494-s001.zip › Supplementary information.pdf]

Freeze-Thaw-Induced Hybrid Porous PVA/PEG Hydrogels with Dynamic Load-Dissipation Capability for Cartilage Substitutes

Luon Tan Nguyen, Patrick Kai Xuan Lim, Wenjuan Jin, Yanli Zheng, Quang M. N. Phan, Meng Wang, Duc Anh Tran, Y. B. Guo, V. P. W. Shim, Huy-Du Do, Thanh-Tan Nguyen, Hieu Tran-Van, Nga H. N. Do, and Hai M. Duong

Supporting information

Table S1. Estimated production cost of PVA/PEG hydrogels

No.	Type of cost	Specifications	Unit	Number	Cost per unit (USD)	Total cost (USD)	Reference source
	**Cost of investing**					**3,585.00**	
1	Agitator		ea	1	2000	2000	Made-in-China
2	Freezer	4 shelves, interior widthxdepth of 100x50 cm	ea	1	1500	1500	WebstaurantStore
3	Trays	Stainless steel, 50x30x2.5 cm	ea	12	7	85	Amazon
	**Cost of operating**					**1,890.40**	
4	PVA	Purity > 99 %	kg	4	263	1052	Sigma-Aldrich
5	PEG	BioUltra	kg	4	186	744	Sigma-Aldrich
6	Water	Distillation	L	20	4	80	ChemWorld.com
7	Cost of energy	Commercial price	kWh	60	0.24	14.40	US Energy Information Administration
	**Equipment depreciation per batch[a]**					**3.59**	
	**Labor cost per batch [b]**					**372.60**	
	**Final total cost**					**2,266.59**	

[a] Equipment depreciation is calculated for 1000 batches of production

[b] Labor cost is estimated as 25 % of operating cost

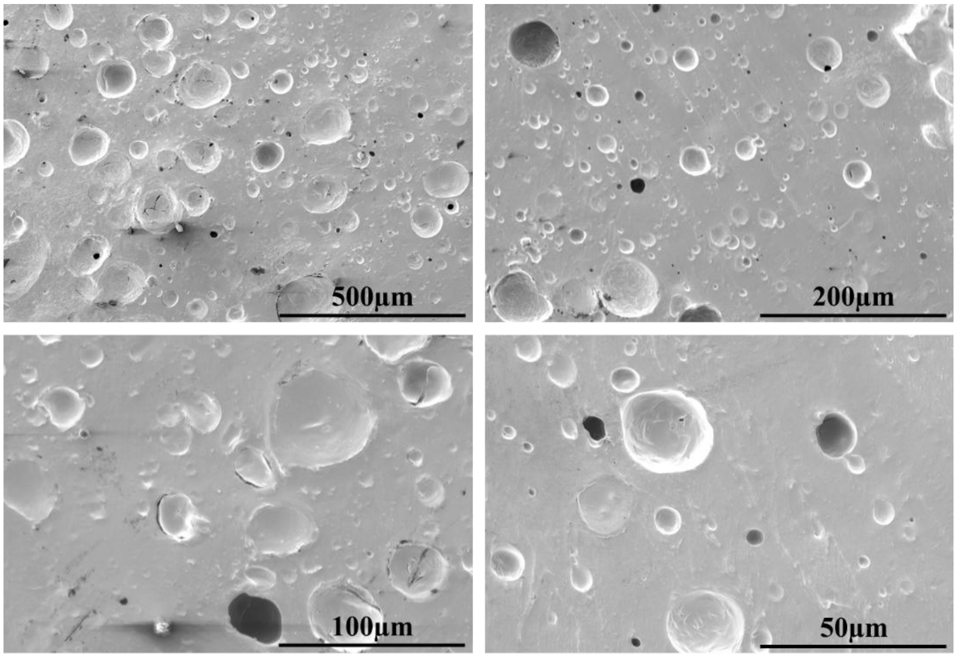

Figure S1. SEM images showing morphological characteristics and porous microstructure of PVA/PEG hydrogels (PPH2) at different magnifications.
